# Editorial: Measurable residual disease in hematologic malignancies

**DOI:** 10.3389/fonc.2023.1204393

**Published:** 2023-05-10

**Authors:** Francesco Buccisano, Raffaele Palmieri, Monica L. Guzman, Sara Galimberti

**Affiliations:** ^1^ Department of Biomedicine and Prevention, University of Rome Tor Vergata, Rome, Italy; ^2^ Weill Cornell Medicine, Cornell University, White Plains, NY, United States; ^3^ Division of Hematology, University Hospital of Pisa, Pisa, Italy

**Keywords:** MRD - measurable residual disease, multiparameter flow cytometry, polymerase chain reaction, surrogate endpoint biomarker, next generation sequencing, next generation flow cytometry (NGF), chimerism after allo-HSCT

Measurable residual disease (MRD) has progressively taken a central role in the field of hematological malignancies, not only as a reliable marker of quality of response to treatment but also as a guide for the decision-making therapeutic choice. Accordingly, MRD is increasingly incorporated in experimental trials and in daily clinical practice, potentially representing a surrogate biomarker to accelerate drug development and approval.

In chronic (Benintende et al.; Robak and Robak) and acute (Tettero et al.) blood malignancies, either in the adult or in the pediatric (Lee et al.) setting, MRD can be considered a reliable prognostic biomarker in that it can provide an estimate of clinical response (Visentin et al.). This information may be particularly relevant in those hematological malignancies that are inherently characterized by a high risk of recurrence, such as acute leukemias (Chiusolo et al., Malagola et al.). In this subset, MRD might be incorporated into clinical trials as a “therapeutic target” to reduce disease burden before curative-intended strategies (including hematopoietic stem cells transplantation) or to indicate treatment de-intensification to spare unnecessary toxicities in patients with no evidence of residual disease (Tecchio et al.). A potential consequence of this assessment is that it may eventually improve the clinical outcome and also have a favorable impact on the financial cost of the overall treatment strategy. Indeed, the probability of hospitalizing patients achieving MRD negativity is usually lower, just as their inpatient stay is shorter.

Besides giving a reliable estimate of the quality of response, MRD monitoring after treatment may also allow to significantly shorten the time to new drug approval if validated as a surrogate endpoint for overall and disease-free survival. Based on this, the U.S Food and Drug Administration (FDA) has recently released a guidance document for the use of MRD in clinical trials testing new drugs for approval. According to this document, the assumption that MRD negativity correlates with a relatively small amount of residual cancer cells, thus representing a “biologically plausible” surrogate for a longer survival, should be actively pursued in clinical trials. Therefore, several trials are now incorporating MRD as an endpoint to accelerate new drug testing and approval, particularly in acute and chronic lymphocytic leukemia and multiple myeloma.

From the technical standpoint, the innate heterogeneous nature of hematological malignancies has prompted the improvement of the sensitivity and specificity of the available techniques and also the design of new tools to track cancer populations more efficiently than “standard” MRD might do. This may be particularly relevant for the identification of cancer stem cells, which are thought to be responsible for disease relapse in those cases of apparent MRD negativity, or the refinement of post-transplant chimerism assessment to identify an impending relapse. Similarly, there is accumulating evidence on the role of next-generation sequencing (NGS) and digital polymerase chain reaction (PCR)-based techniques for MRD determination in addition to reverse transcriptase quantitative PCR and multiparametric flow cytometry (Pacelli et al.; Assanto et al.). In which type of disease these novelties will become stand-alone techniques is not yet known.

Irrespective of the clinical subset and of the source to be tested for MRD (peripheral blood rather than bone marrow or other tissues), assessing the quality of the sample is critical to ensure the reliability of the assay. This can be particularly critical when testing the bone marrow, since either the background noise due to normal hematopoiesis or the poor quality of samples (e.g., hemodilution) can significantly reduce the sensitivity and specificity of the tests (Vigliotta et al.). However, since no consensus has been established yet on criteria for samples’ quality acceptability, specific guidelines to address this issue are needed in the near future.

In conclusion, thanks to the accumulating evidence from prospective and retrospective MRD-centered trials, the “why” of testing MRD (e.g., the development of MRD-driven strategies) is becoming progressively clear. Nevertheless, until harmonization–standardization efforts are accomplished by the scientific community, “who” (e.g., patients that may benefit from testing), “what” (e.g., which biomarkers are suitable for MRD monitoring), “where” (e.g., which is the optimal source for MRD monitoring), “when” (e.g., which timepoints are crucial for clinical decision-making), and “how” (e.g., which technique is fit for which patient) to assess MRD will remain open questions (**Figure 1**).

**Figure 1:**
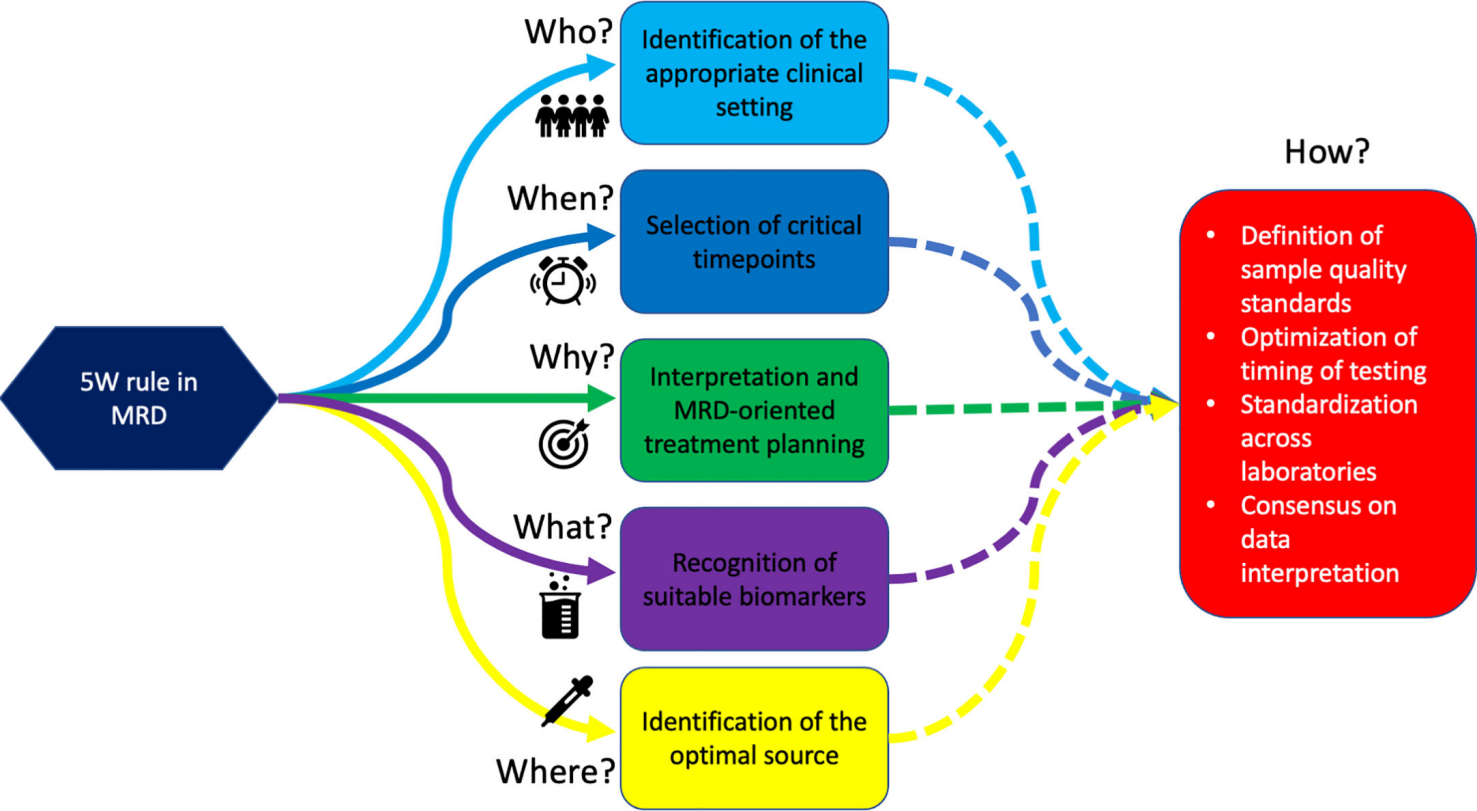
The 5W rules in MRD. We are progressively learning *Why* test to MRD, *Who* deserves MRD monitoring, *Which* population should be monitored, *Where* to search for MRD, and *When* to assess it. This will eventually lead to defining “How” we should do it.

## Author contributions

FB and RP wrote the manuscript and drawn the figure, MG and SG critically reviewed the test. All authors contributed to manuscript revision, read, and approved the submitted version.

